# Is extraordinary prosocial behavior more valuable than ordinary prosocial behavior?

**DOI:** 10.1371/journal.pone.0196340

**Published:** 2018-04-23

**Authors:** Ikumi Futamura

**Affiliations:** Graduate School of Education and Human Development, Nagoya University, Furo-cho, Chikusa-ku, Nagoya, Japan; University of the Basque Country, SPAIN

## Abstract

This study examined how people evaluate ordinary and extraordinary prosocial behaviors, especially their predictions of the likelihood of future prosocial behaviors of ordinary and extraordinary prosocial actors (Study 1). Further, it examined the individual effects of ordinary and extraordinary prosocial behaviors of an actor on the evaluation of his/her trait by considering the cases where the actor engages in and does not engage in the other behavior (Study 2). Study 1 revealed that the likelihood of future prosocial behaviors of ordinary and extraordinary prosocial actors was perceived asymmetrically. Specifically, while the likelihood of ordinary prosocial actors to engage in ordinary prosocial behaviors was perceived as high, the same perception was not observed for extraordinary prosocial behaviors. On the other hand, extraordinary prosocial actors were perceived as highly likely to engage in both ordinary and extraordinary prosocial behaviors. Study 2 revealed that the evaluation of actors who engaged in extraordinary prosocial behaviors but not ordinary prosocial behaviors did not exceed the evaluation of actors who engaged in ordinary prosocial behaviors but not extraordinary prosocial behaviors. Additionally, the effect of extraordinary prosocial behaviors was more when the actor also engaged in ordinary prosocial behaviors. These results suggest that extraordinary prosocial actors are evaluated highly when they also engage in ordinary prosocial behaviors.

## Introduction

Prosocial behaviors are necessary for human society. It is important for the maintenance of prosocial behaviors in society that prosocial actors be perceived as helpful individuals because that increases the actors’ chances of receiving help from others [1–2]. However, such behaviors appear in a variety of forms, and cognition regarding them may differ depending on their features.

In *Theory of Moral Sentiments* of Adam Smith, he differentiated between “ordinary” prosocial behavior and “extraordinary” prosocial behavior [3]. Ordinary prosocial behaviors are those behaviors involving relatively high situational and sociocultural demands, the need for which is clear, and in which relatively large numbers of people commonly engage, for example, the reciprocal prosocial behavior of helping behavior after the actor had received help from that person and prosocial behavior under a role constraint. On the other hand, extraordinary prosocial behaviors are those behaviors that have relatively low situational and sociocultural demands, the need for which is sometimes ambiguous, and in which fewer people engage, such as the unilateral prosocial behavior of helping behavior after the actor’s own request for help had not been accepted by that person or prosocial behavior without role constraints.

Earlier studies in developmental psychology have indicated that adults evaluated extraordinary prosocial actors more positively than ordinary prosocial actors. For instance, it was found that adult participants evaluated a unilateral prosocial actor who had helped a person after the actor’s own request for help had not been accepted by that person higher than a reciprocal prosocial actor who had helped an individual after receiving help from that individual [4–6].

In contrast, several social psychological studies have recently reported that the evaluations of extraordinary prosocial actors by others did not vary from those of ordinary prosocial actors [7–9]. For example, Experiment 1a in the study by Klein and Epley [8] found that participants did not judge a concert-goer who donated more than the suggested donation amount more positively than a person who donated only the suggested amount. This result seems to indicate that extraordinary prosocial behaviors do not have extra value over ordinary prosocial behaviors.

Based on the points discussed above, the findings pertaining to the cognition of ordinary and extraordinary prosocial behaviors seem contradictory and ambiguous. It is important to examine cognition concerning such actions in order to improve our understanding of the social functions of such prosocial behaviors. To gain more insight on this issue, Futamura [10] examined the evaluation of ordinary and extraordinary prosocial actors by focusing on the experimental design and measures used for evaluation, but several questions remain unexamined. The aim of the present study was to examine the questions that have not yet been dealt with, which are the prediction of the likelihood of future prosocial behaviors of ordinary and extraordinary prosocial actors (Study 1) and the effects of ordinary and extraordinary prosocial behaviors on trait evaluation in the cases of the actor engaging in and not engaging in the other behavior (Study 2).

### Trait evaluation of ordinary and extraordinary prosocial actors

The main differences in the earlier studies that yielded apparently contradictory results pertain to the experimental design and the measures used for evaluation. Importantly, the studies that reported that participants evaluated extraordinary prosocial actors more positively than ordinary ones used a within-participants design, while the studies showing no differences between the evaluations of the two groups used a between-participants design. Experiment 4 of Klein and Epley [8] compared the evaluations of donors who donated different amounts of money using a within-participants design and a between-participants design, which showed that more generous actions were evaluated increasingly positively in the within-participants design, but that in the between-participants design the participants did not think that giving more money was increasingly positive. This result could be interpreted as suggesting that people might evaluate extraordinary prosocial actors more positively than ordinary prosocial actors only when using a within-participants design.

However, Futamura [10] assumed that the difference in evaluations between ordinary and extraordinary prosocial actors was not merely an artifact of the use of a within-participants design, but rather claimed that a difference would be detected even with a between-participants design if one uses appropriate measures for evaluation. The measures used for evaluation in previous studies have varied widely, including kindness [4], the number of nickels for the person who does the nicest things [5], and traits of “warmth” based on the fundamental dimensions identified by Fiske, Cuddy, Glick, and Xu [11] (Experiment 1a of [8]). However, recent studies regarding individual perceptions suggest that social goodness, which was regarded as the sole dimension of “warmth” in such previous studies as Fiske et al. [11], has two dimensions, morality and warmth, in the narrow sense, and that these two forms of social goodness should be distinguished [12–16]. Morality in the narrow sense is the dimension pertaining to “being benevolent to people in ways that facilitate correct and principled relations with them,” while warmth in the narrow sense is the dimension of “being benevolent to people in ways that facilitate affectionate relations with them” [12]. Note that some studies (e.g., [16]) express warmth as sociability, but in the present paper all such expressions were unified into warmth. Although the measures of kindness and niceness that were used in the studies of Baldwin and Baldwin [4] and Leahy [5] were closer to warmth than morality, the measure of “warmth” used in Klein and Epley’s study [8] integrated morality and warmth. It is possible that these differences in the conceptualization of measures caused the inconsistency in the results of these studies.

Futamura [10] then examined the evaluation of ordinary and extraordinary prosocial actors with a between-participants design using the measures of morality and warmth that were used in the study by Leach et al. [16], and the concepts of those measures corresponded to the descriptions about morality and warmth in the narrow sense in the study by Brambilla and Leach [12]. The measures of morality and warmth in Leach et al. [16] consisted of the following three items each: “honest,” “sincere,” and “trustworthy” for morality and “likeable,” “warm,” and “friendly” for warmth. This study found no difference between the evaluations of ordinary and extraordinary prosocial actors with respect to morality, but the latter were evaluated more highly with respect to warmth. This result suggests that ordinary and extraordinary prosocial actors are not evaluated differently simply due to use of a within-participants design, but rather that people evaluate them differently depending on the dimensions of social goodness under consideration. The findings indicate that extraordinary prosocial actors were not necessarily evaluated higher than ordinary prosocial actors in all dimensions; however, they were evaluated higher particularly in the dimension of forming affectionate relationships with others. This result provided a framework that facilitates an integrated understanding of contradictory findings of previous studies.

### Prediction of behavior of ordinary and extraordinary prosocial actors

However, there is still a lack of understanding of the cognition of ordinary and extraordinary prosocial behaviors. One point that should be examined further is predictions of the future behavior of ordinary and extraordinary prosocial actors. Lin-Healy and Small [17] reported a helpful finding with respect to this topic. They compared the perceived likelihood of future prosocial behaviors of two types of donors to a charity, one with a personal facilitatory cause for the donation and the other without such a cause. The results showed that the donor without any facilitatory cause was perceived as more likely to engage in future prosocial behaviors than was the donor with a personal facilitatory cause. Based on this finding, it was predicted that the likelihood of future prosocial behaviors of extraordinary prosocial actors is perceived as higher than that of ordinary prosocial actors.

However, Lin-Healy and Small [17] also showed that the donor without any facilitatory cause was perceived as more likely to engage in future prosocial behaviors in unrelated domains, but not with the same target charity. Therefore, how people perceive the differences in the likelihood of future prosocial behaviors by ordinary and extraordinary prosocial actors might also differ depending on the features of the future prosocial behaviors that are the targets of the prediction. Skowronski and Carlston [18] argued that extreme behaviors are characteristic of only those who have extreme scores on the pertinent trait dimensions, whereas moderate behaviors may be characteristic of those who have either moderate or extreme scores on the pertinent trait dimension. Based on this proposition, it is predicted that people perceive the traits of ordinary and extraordinary prosocial actors differently and infer the likelihood of their engagement in future ordinary and extraordinary prosocial behaviors differently. An asymmetric inference about the future behavior prediction of ordinary and extraordinary prosocial actors is expected as follows. The likelihood of ordinary prosocial actors to engage in ordinary prosocial behaviors is perceived as high, but that of engaging in extraordinary prosocial behaviors is not necessarily perceived as high. On the other hand, extraordinary prosocial actors’ likelihood to engage in both ordinary and extraordinary prosocial behaviors is perceived as high.

### The effects of ordinary and extraordinary prosocial behaviors on trait evaluation

As discussed above, if the extraordinary prosocial actors are perceived as highly likely to also engage in ordinary prosocial behaviors, it is assumed that people perceive it as natural for extraordinary prosocial actors to also engage in ordinary prosocial behaviors. It is predicted that the information about engaging in ordinary prosocial behaviors does not substantially affect the evaluation of extraordinary prosocial actors and that people evaluate the person who engages in an extraordinary prosocial behavior as almost the same as the actor who engages in both ordinary and extraordinary prosocial behaviors. Consequently, we need to examine how engaging in extraordinary prosocial behaviors affects trait evaluation of the person who does not engage in ordinary prosocial behaviors. Another question is, how the effect of engaging in extraordinary prosocial behaviors is different in the case of the person engaging in an ordinary prosocial behavior and in the case of the person not engaging in that. To understand the function of ordinary and extraordinary prosocial behaviors on trait evaluation, it is considered important to examine not only the evaluation of actors with only the information about engagement in either ordinary or extraordinary prosocial behaviors but also the evaluation of actors whose patterns of behaviors consist of a combination of engagement or non-engagement in ordinary and extraordinary prosocial behaviors. By systematically manipulating engagement or non-engagement of both behaviors simultaneously, this study examines the effects of ordinary and extraordinary prosocial behaviors on trait evaluation of the actors to promote a better understanding of how these behaviors affect trait evaluation.

## Study 1

Study 1 examined predictions of future prosocial behavior by ordinary and extraordinary prosocial actors. It was predicted that the likelihood of ordinary prosocial actors to engage in ordinary prosocial behaviors is perceived as high but that of engaging in extraordinary prosocial behaviors is not necessarily perceived as high. On the other hand, the likelihood of extraordinary prosocial actors to engage in both ordinary prosocial behaviors and extraordinary prosocial behaviors is perceived as high.

In addition, a limitation of Futamura [10] is that only a few items of the questionnaire used assessed morality and warmth. Therefore, the present Study 1 examined trait evaluation using two additional measures each of morality and warmth to confirm whether the results of Futamura [10] are replicated. Regarding trait evaluation, based on Futamura [10], it was predicted that there would be no differences in the evaluations of ordinary and extraordinary prosocial actors with respect to morality; however, with respect to warmth, extraordinary prosocial actors would be evaluated more highly than ordinary prosocial actors.

### Method

#### Participants and procedure

A total of 112 university students participated in this online study, and they received course credit for their participation. Their informed consent was obtained by asking them to check the related item on the online page. This study included minors, but the need for consent from parents or guardians was waived by the ethics committee. Three participants did not answer one or more of the questions and six participants failed to give correct answers to items used to confirm their understanding of the scenarios, resulting in a final sample of 103 participants (40 males, 63 females, *M*_age_ = 18.54 years, *SD* = 0.88 years; one participant did not report age). Participants were randomly assigned either to the condition in which they read scenarios relating to ordinary prosocial actors (OR condition) or to that relating to extraordinary prosocial actors (EX condition).

#### Scenarios

Ordinary and extraordinary prosocial behaviors are concepts that include a range of behaviors. Therefore, two scenarios were used for each condition to avoid the results from being influenced too strongly by the individual scenario settings and to enhance the validity of discussions of ordinary and extraordinary prosocial behaviors. In the first of the two scenarios, based on Baldwin and Baldwin [4], Suls et al. [6], and Futamura [10], the ordinary prosocial behavior was a reciprocal behavior, while the extraordinary prosocial behavior was a unilateral behavior (reciprocity scenario). Specifically, in the OR condition, the scenario involved a reciprocal prosocial actor who lent a notebook to a person after having borrowed a notebook from that same person. In the EX condition, the scenario involved a unilateral prosocial actor who lent a notebook to a person after the actor’s request to borrow a notebook from that same person had not been accepted. In the second scenario, based on Baldwin and Baldwin [4], the ordinary prosocial behavior was a prosocial behavior under a role constraint, while the extraordinary prosocial behavior was a prosocial behavior without a role constraint (role scenario). Here, in the OR condition, the scenario described a prosocial actor who showed the way to a person who had lost his/her way while touring the campus in a situation in which the prosocial actor was an official guide for the campus tour. In the EX condition, the scenario described a prosocial actor who acted in the same way (i.e., showed the way) but was merely a passerby. As a control, all of the characters were described as “the same sex as you (the participant).” The order of presentation of the reciprocity and role scenarios was counterbalanced. Additionally, some items tested the participants’ understanding of the scenarios.

#### Manipulation check

As a manipulation check of the degree of situational demand for the prosocial behaviors described in the scenarios, participants’ perception of the need for the protagonist to engage in the described prosocial behavior was assessed with the following two items using a 7-point Likert scale: “How necessary was it for A to (perform)…for B in this situation? (1 = *not necessary at all*, 7 = *absolutely necessary*)”; and “Do you think A should (perform)…for B in this situation? (1 = *does not have to…at all*, 7 = *absolutely should…*)”.

#### Trait evaluation

Participants were presented a list of five morality traits (honest, sincere, trustworthy, respectful, righteous) and five warmth traits (likable, warm, friendly, kind, helpful), and they evaluated the relevance of these traits on a 7-point Likert scale (1 = *not at all*, 7 = *very much*). These items were used in Brambilla et al.’s study [13].

#### Behavior prediction

Using a 7-point Likert scale, participants were asked to indicate the protagonist’s likelihood to engage in both ordinary and extraordinary prosocial behaviors. The ordinary and extraordinary prosocial behaviors were the same as those in the OR and EX conditions, but the target persons of the prosocial behaviors were different.

### Results

In the reciprocity and role scenarios, the Cronbach’s α for the five items related to morality was .72 and .82, respectively, and that for the five items related to warmth was .90 and .88, respectively. Because there was no salient difference between the trends of results for the reciprocity and role scenarios, scores were averaged across these two scenarios in subsequent analyses.

#### Manipulation check

The averages of the two items relating to the perception of the need for the behavior were compared for the OR and EX conditions. The perception of the need in the OR condition (*M* = 5.80, *SD* = 0.57) was higher than in the EX condition (*M* = 4.19, *SD* = 0.90), with *t* (90) = 10.89, *p* < .001, Cohen’s *d* = 2.13.

#### Trait evaluation

The averages and *SDs* of the morality and warmth measures in the OR and EX conditions are shown in Table 1. As indicated, there was no difference between the OR and the EX conditions with respect to morality (*t* (101) = 0.89, *p* = .38), but the warmth value in the EX condition was greater than that in the OR condition (*t* (101) = 4.26, *p* < .001, Cohen’s *d* = 0.85).

**Table 1:** Mean Ratings (and Standard Deviations) of the trait evaluation of the ordinary and extraordinary prosocial actors.

	Conditions	
Trait evaluation	OR	EX
Morality	5.06 (0.74)	5.19 (0.74)
Warmth	5.46 (0.74)	6.08 (0.73)

#### Behavior prediction

The averages and *SDs* of the prediction of the protagonist’s likelihood to engage in ordinary and extraordinary prosocial behaviors are shown in Table 2. A 2 (condition: OR or EX) × 2 (target behavior of the prediction: ordinary or extraordinary prosocial behavior) mixed ANOVA for the scores of perceived likelihood of engaging in each prosocial behavior revealed a significant interaction effect (*F* (1, 101) = 34.32, *p* < .001, $\eta_{p}^{2}$ = 0.25). While the actors in the EX condition were perceived as more likely to engage in both ordinary (*F* (1, 101) = 19.90, *p* < .001, $\eta_{p}^{2}$ = 0.16) and extraordinary prosocial behaviors (*F* (1, 101) = 75.74, *p* < .001, $\eta_{p}^{2}$ = 0.43) than were those in the OR condition, the difference between the OR and EX conditions was larger in the perceived likelihood of extraordinary prosocial behaviors than it was in the likelihood of ordinary prosocial behaviors. The simple main effects of the target behavior of the prediction were also significant for both conditions: OR condition (*F* (1, 48) = 241.34, *p* < .001, $\eta_{p}^{2}$ = 0.83), EX condition (*F* (1, 53) = 97.39, *p* < .001, $\eta_{p}^{2}$ = 0.65).

**Table 2:** Mean Ratings (and Standard Deviations) of the prediction of the prosocial behavior of ordinary and extraordinary prosocial actors.

	Conditions	
Target behavior of the prediction	OR	EX
Ordinary prosocial behavior	6.26 (0.66)	6.75 (0.45)
Extraordinary prosocial behavior	4.00 (0.97)	5.58 (0.87)

### Discussion

Regarding trait evaluation, the result of Futamura [10] was replicated by the current Study 1; while there was no difference between the OR and EX conditions with respect to morality, warmth was evaluated more highly in the EX condition than it was in the OR condition. Regarding behavior prediction, while extraordinary prosocial actors’ likelihood to engage in ordinary and extraordinary prosocial behaviors and ordinary prosocial actors’ likelihood to engage in ordinary prosocial behavior were perceived as high, ordinary prosocial actors’ likelihood to engage in extraordinary prosocial behavior was just around the midpoint of the 7-point scale. This indicates that people assess the likelihood of different behaviors of ordinary and extraordinary prosocial actors asymmetrically; they consider it highly likely for an extraordinary prosocial actor to engage in ordinary prosocial behaviors, but they assign a lower likelihood for the ordinary prosocial actor to exhibit extraordinary prosocial behaviors.

## Study 2

Study 2 examined the effects of ordinary and extraordinary prosocial behaviors on trait of actors in cases of patterns of behaviors as a 2 (ordinary prosocial behavior: engaging or non-engaging) × 2 (extraordinary prosocial behavior: engaging or non-engaging) combination.

### Method

#### Participants and procedure

A total of 405 university students participated in the online study. Some participants received course credit by participating. Others were recruited through class announcements, and they participated in good faith without compensation. Informed consent was obtained from all the participants by asking them to check the related item on the online page. This study included minors, but the need for consent from parents or guardians was waived by the ethics committee. Thirteen participants did not provide answers to one or more of the questions and 26 participants gave incorrect answers to items testing their understanding of the scenarios, resulting in a final sample of 366 participants (146 males, 220 females, *M*_age_ = 19.08 years, *SD* = 1.49 years). Participants were randomly assigned to one of six conditions in which participants read scenarios concerning (a) actors who engaged in both ordinary and extraordinary prosocial behaviors (OR-EX condition), (b) actors who engaged in an ordinary prosocial behavior but not in an extraordinary prosocial behavior (OR-ex condition), (c) actors who did not engage in an ordinary prosocial behavior but engaged in an extraordinary prosocial behavior (or-EX condition), (d) actors who engaged in neither ordinary nor extraordinary prosocial behaviors (or-ex condition), (e) actors who engaged in ordinary prosocial behavior (OR condition), and (f) actors who engaged in extraordinary prosocial behavior (EX condition). It should be noted that the OR and EX conditions were the same as those used in Study 1 so as to allow for comparison with the OR-EX condition. The conditions employed in Study 2 are summarized in Table 3.

**Table 3:** Summary of the conditions in Study 2.

	1. OR-EX	2. OR-ex	3. or-EX	4. or-ex	5. OR	6. EX
Ordinary prosocial behavior	1	1	0	0	1	-
Extraordinary prosocial behavior	1	0	1	0	-	1

“1” indicates “engaging” in prosocial behavior; “0” indicates “not engaging” in prosocial behavior; “-” indicates “no information” about engaging in prosocial behavior.

#### Scenarios

As in Study 1, each condition included two scenarios, a reciprocity scenario and a role scenario. For example, the reciprocity scenario in the OR-EX condition described a situation in which the actor lends a notebook to another person after the same person did not agree to lend a notebook to the actor; the scenario has the same actor also lending a notebook to another person after the actor had borrowed a notebook from that person in another situation. As a control, the sex of the characters in each of the scenarios was described as “the same sex as you.” The order of presentation of the reciprocity and role scenarios was counterbalanced in all conditions, and the order of presentation of ordinary and extraordinary prosocial behaviors was counterbalanced in the OR-EX, OR-ex, or-EX, and or-ex conditions. Participants were asked to answer items designed to test their understanding of the scenarios.

#### Manipulation check

In each of the conditions, the participants’ perception of the need to engage in the behaviors described in the scenarios was assessed on a 7-point Likert scale using the following item: “How necessary was it for A to (perform)…for B in this situation? (1 = *not necessary at all*, 7 = *absolutely necessary*)”.

#### Trait evaluation

Participants evaluated the relevance of each trait on a 7-point Likert scale (1 = *not at all*, 7 = *very much*), as in Study 1.

### Results

In the reciprocity and role scenarios, the Cronbach’s α for the five items related to morality was .89 and .92, respectively, and that for the five items related to warmth was .97 and .98, respectively. As in Study 1, scores were averaged across the scenarios in subsequent analyses.

#### Manipulation check

The perception of the need for the behavior in the scenarios was compared by conducting a paired *t* test on the average scores in the OR-EX, OR-ex, or-EX, and or-ex conditions, since each participant assessed the need for both, the ordinary as well as the extraordinary prosocial behavior. For the OR and EX conditions, an unpaired *t* test was conducted since in these cases each participant assessed only one of the behaviors. The results showed that for the OR-EX, OR-ex, or-EX, and or-ex conditions, the need for the ordinary prosocial behavior (*M* = 6.18, *SD* = 0.60) was evaluated more highly than was the need for the extraordinary prosocial behavior (*M* = 3.94, *SD* = 1.06; *t* (240) = 32.41, *p* < .001, Cohen’s *d* = 2.08). In addition, for the OR and EX conditions the need for the ordinary prosocial behavior (*M* = 5.85, *SD* = 0.90) was evaluated more highly than the need for the extraordinary prosocial behavior (*M* = 3.71, *SD* = 0.98), with *t* (123) = 12.67, *p* < .001, and Cohen’s *d* = 2.29.

#### Trait evaluation

**Fig 1:**
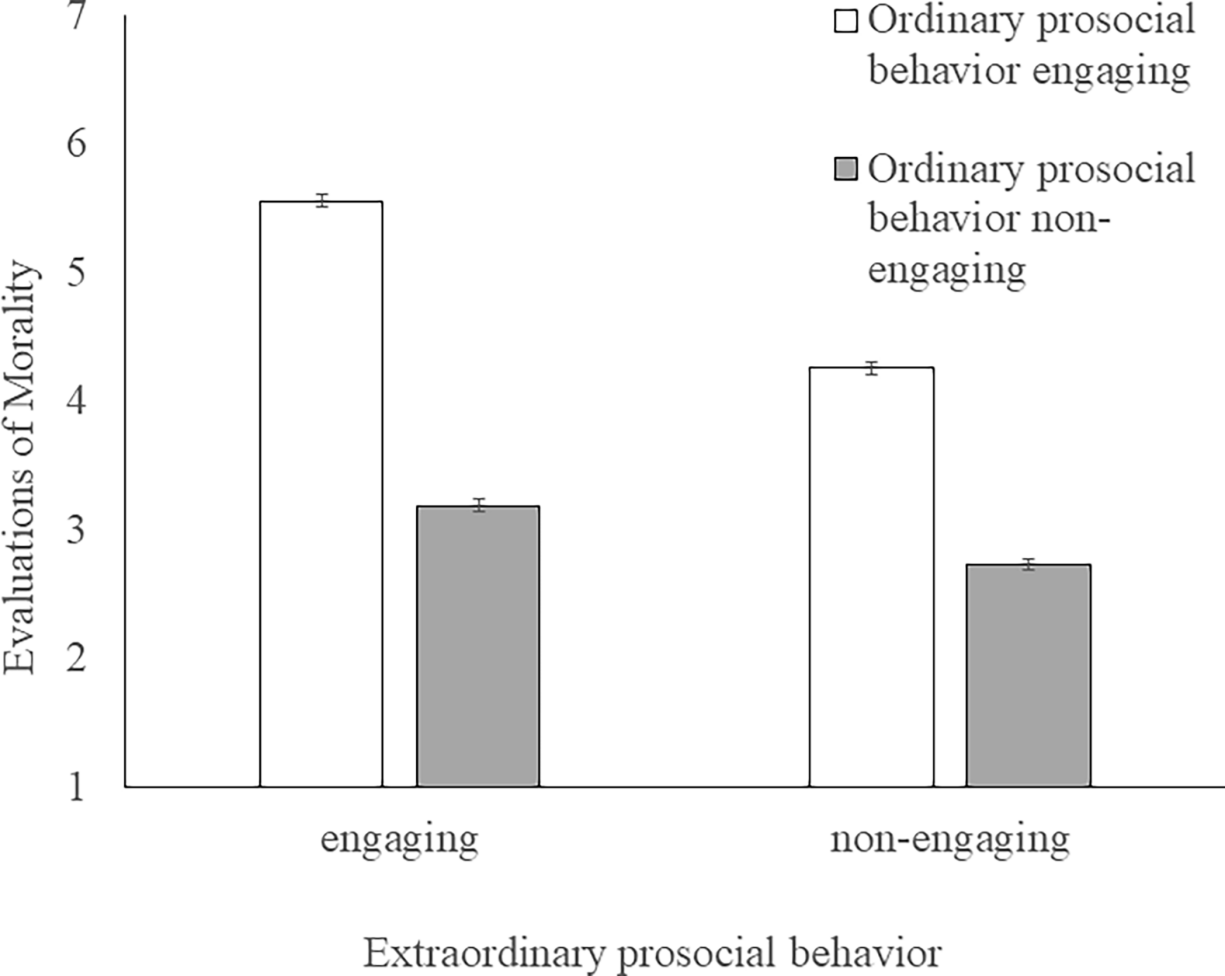
The influence of acting or Non-Acting of ordinary and extraordinary prosocial behaviors on the evaluation of morality. Error bars represent standard errors.

**Fig 2:**
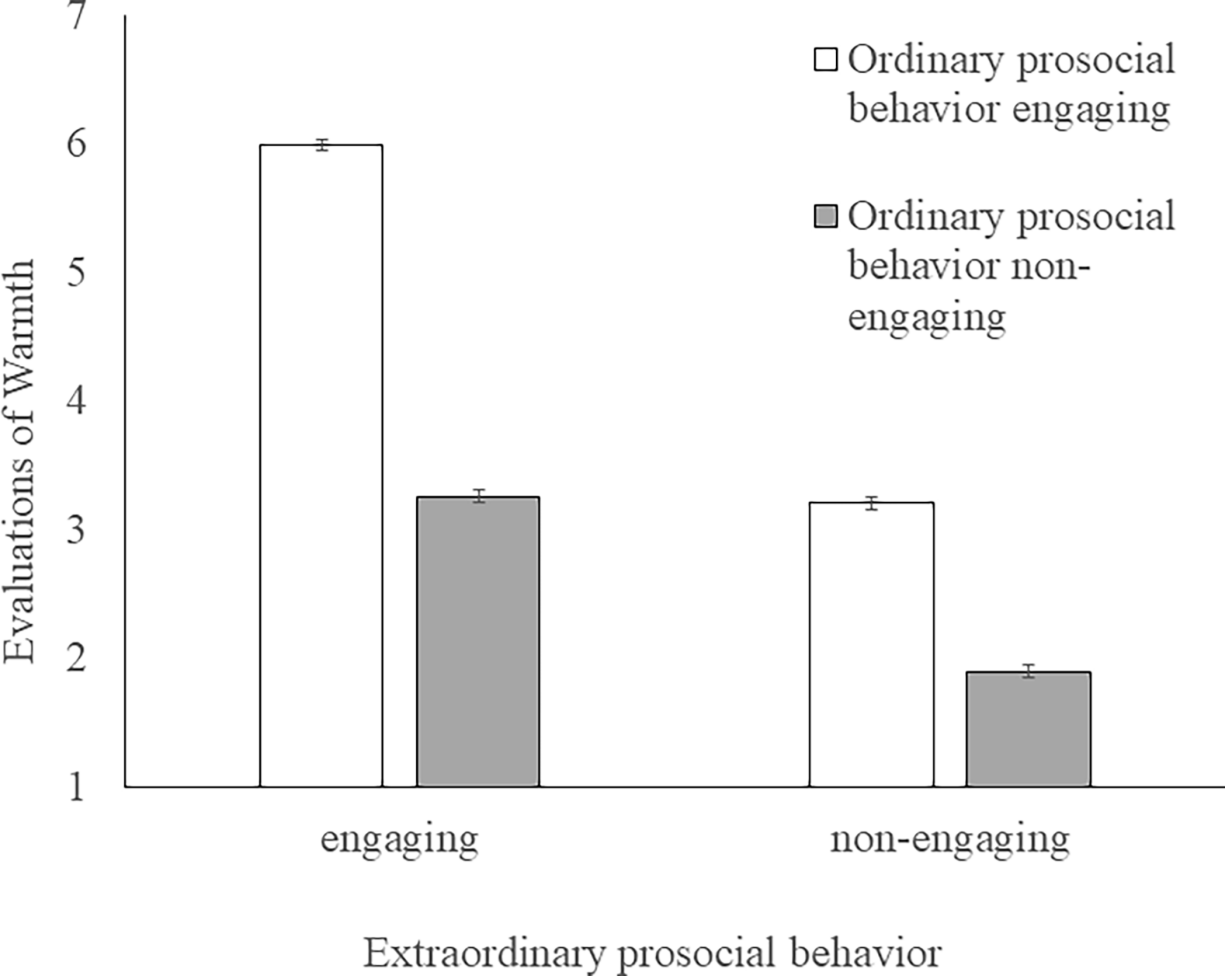
The influence of acting or Non-Acting of ordinary and extraordinary prosocial behaviors on the evaluation of warmth. Error bars represent standard errors.

The averages and *SDs* for morality and warmth in each condition are shown in Table 4. A 2 (ordinary prosocial behavior: engaging or non-engaging) × 2 (extraordinary prosocial behavior: engaging or non-engaging) between-participant ANOVA for morality revealed a significant interaction effect (*F* (1, 237) = 20.04, *p* < .001, $\eta_{p}^{2}$ = 0.08, Fig 1). The simple main effects of ordinary prosocial behavior were also significant on both engaging in extraordinary prosocial behavior (*F* (1, 237) = 335.79, *p* < .001, $\eta_{p}^{2}$ = 0.59) and not engaging in extraordinary prosocial behavior (*F* (1, 237) = 124.87, *p* < .001, $\eta_{p}^{2}$ = 0.35). The simple main effects of extraordinary prosocial behavior were significant both on engaging in ordinary prosocial behavior (*F* (1, 237) = 92.82, *p* < .001, $\eta_{p}^{2}$ = 0.28) and not engaging in ordinary prosocial behavior (*F* (1, 237) = 11.97, *p* < .001, $\eta_{p}^{2}$ = 0.05). The same ANOVA for warmth also revealed a significant interaction effect (*F* (1, 237) = 52.98, *p* < .001, $\eta_{p}^{2}$ = 0.18, Fig 2). The simple main effects of ordinary prosocial behavior were significant for both engaging in extraordinary prosocial behavior (*F* (1, 237) = 409.84, *p* < .001, $\eta_{p}^{2}$ = 0.63) and not engaging in extraordinary prosocial behavior (*F* (1, 237) = 83.43, *p* < .001, $\eta_{p}^{2}$ = 0.26). The simple main effects of extraordinary prosocial behavior were significant for both engaging in ordinary prosocial behavior (*F* (1, 237) = 391.44, *p* < .001, $\eta_{p}^{2}$ = 0.62) and not engaging in ordinary prosocial behavior (*F* (1, 237) = 96.96, *p* < .001, $\eta_{p}^{2}$ = 0.29).

**Table 4:** Mean Ratings (and Standard Deviations) of the trait evaluation in each condition.

	Conditions						Multiple comparisons
Trait evaluation	1. OR-EX	2. OR-ex	3. or-EX	4. or-ex	5. OR	6. EX	
Morality	5.56 (0.70)	4.26 (0.71)	3.19 (0.78)	2.74 (0.72)	5.57 (0.70)	5.31 (0.80)	1 > 2, 1 > 3, 1 > 4, 2 > 3, 2 > 4, 5 > 2, 5 > 3, 5 > 4, 6 > 2, 6 > 3, 6 > 4 (*p* < .001) 3 > 4 (*p* < .01)
Warmth	5.99 (0.75)	3.21 (0.72)	3.26 (0.79)	1.91 (0.78)	5.58 (0.82)	6.04 (0.85)	1 > 2, 1 > 3, 1 > 4, 2 > 4, 3 > 4, 5 > 2, 5 > 3, 5 > 4, 6 > 2, 6 > 3, 6 > 4 (*p* < .001) 1 > 5, 6 > 5 (*p* < .01)

The influences of the six conditions, including the OR and EX conditions relating to morality and warmth, were examined. One-way ANOVAs revealed a significant effect of condition on morality (*F* (5, 360) = 177.11, *p* < .001, *η*^2^ = 0.71) and warmth (*F* (5, 360) = 306.84, *p* < .001, *η*^2^ = 0.81). The results of multiple comparisons using the Tukey-Kramer method are shown in Table 4. There was no difference between the OR-ex and or-EX conditions with respect to warmth; however, the evaluation of morality in the OR-ex condition was higher than in the or-EX condition. In the OR-EX, OR, and EX conditions, there were no differences in the evaluation of morality; however, the evaluation of warmth in the OR-EX and EX conditions was higher than that in the OR condition. There was no difference between the OR-EX and EX conditions for either morality or warmth.

### Discussion

Study 2 revealed a significant interaction effect and simple main effects for ordinary and extraordinary prosocial behaviors on both morality and warmth. In addition, the results indicate that the effect of extraordinary prosocial behavior on trait evaluation was greater when the actor also engaged in ordinary prosocial behavior. Furthermore, the evaluation of the or-EX actor did not exceed the evaluation of OR-ex actor, and, with respect to morality, the OR-ex actor was evaluated higher than the or-EX actor. These results suggest that extraordinary prosocial behaviors are evaluated highly when the extraordinary prosocial actor also engages in ordinary prosocial behavior. Further, the evaluation of morality in either the OR-EX condition or the EX condition did not exceed that in the OR condition; however, the evaluation of warmth in the OR-EX and EX conditions was higher than that in the OR condition. These results suggest that engaging in extraordinary prosocial behaviors has added value when evaluating the warmth of the actor. In addition, there was no difference between the OR-EX and EX conditions in terms of either morality or warmth. This is consistent with the result of Study 1, that is, people infer that the extraordinary prosocial actors also perform ordinary prosocial behaviors.

## General discussion

Study 1 revealed that the likelihood for ordinary prosocial actors to engage in ordinary prosocial behaviors is perceived as high, but that to engage in extraordinary prosocial behaviors is not necessarily perceived as high. On the other hand, extraordinary prosocial actors’ likelihood to engage in both ordinary prosocial behaviors and extraordinary prosocial behaviors is perceived as high. This indicates that people predict the likelihood of future prosocial behaviors of ordinary and extraordinary prosocial actors asymmetrically.

Study 2 revealed that on both dimensions of morality and warmth, the evaluation of the or-EX actor, who did not engage in ordinary prosocial behavior but engaged in extraordinary prosocial behavior, did not exceed that of OR-ex actor, who engaged in ordinary prosocial behavior but did not engage in extraordinary prosocial behavior. However, with reference to morality, the evaluation of the OR-ex actor was higher than that of the or-EX actor. In addition, engaging in extraordinary prosocial behaviors had larger added value when the actors of extraordinary prosocial behaviors would also engage in ordinary prosocial behaviors than when they would not. These results suggest that extraordinary prosocial behaviors are evaluated highly when the extraordinary prosocial actor also engages in ordinary prosocial behaviors. Additionally, the evaluation of warmth in the OR-EX and EX conditions was higher than in the OR condition. This suggests that engaging in extraordinary prosocial behaviors has more added value, especially in the evaluation of warmth.

The difference between morality and warmth described in individual perception studies, such as Brambilla and Leach [12] and Brambilla et al. [13], appears to correspond to the arguments concerning the nature of virtue described in Smith [3] and Hoffman [19]. Smith [3] differentiated between the virtues of justice and beneficence, the former meaning fulfilling an obligation properly and the latter indicating social and benevolent behaviors such as generosity, charity, kindness, and friendship. Hoffman [19] also proposed that justice and caring are often viewed as different moral principles. Smith [3] proposed that justice is the main pillar that upholds the entire edifice of a society, and that beneficence is an ornament that embellishes the comfort of that society. In this way, it is thought that morality reflects the virtues that uphold a society and warmth reflects virtues that contribute to the comfort of that society. Landy et al. [15] proposed that morality and warmth each convey something unique and functionally important about others. Based on the above, the results of the present study suggest that ordinary prosocial behaviors are important for upholding society and extraordinary prosocial behaviors are important for enhancing the comfort of society, and each plays a significant social function.

It was pointed out that the experimental design and measures used for evaluation produced varying results in previous studies that showed no difference between extraordinary and ordinary prosocial actors (e.g., Klein & Epley [8]; Gneezy & Epley [7]). The generality of behaviors might also explain these differences. For example, Experiment 1a in Klein and Epley’s study [8] examined the amount that the study participants donated when attending a concert that was ostensibly free but where concert organizers suggested a certain amount that concertgoers might consider donating. They found that 40% of the participants donated the suggested amount, 50% donated less than the amount, and 10% donated more than the amount. It is not possible to compare the generalities of prosocial behaviors between in the present study and Experiment 1a of Klein and Epley’s study [8] directly because participants’ own behaviors were not measured in the present study. However, both the ordinary and extraordinary prosocial behaviors in the present study did not involve paying any money and the cost for the participants was relatively low. Therefore, it might be possible that ordinary and extraordinary prosocial behaviors in the present study are likely performed by more people than the prosocial behaviors examined by Klein and Epley [8]. Ordinary and extraordinary prosocial behaviors are relative and continuous concepts that cannot be easily and clearly differentiated, and the evaluation of behaviors and actors can be expected to differ as well depending on the generality of those behaviors.

In the present study, the unilateral prosocial behavior, which consisted of helping a person who had not agreed to help, was set as an extraordinary prosocial behavior in the reciprocity scenario based on the studies of Baldwin and Baldwin [4] and Suls et al. [6]. However, helping behavior directed towards a person who has not previously interacted with the giver in any way can also be treated as a unilateral extraordinary prosocial behavior. On this point, Futamura [20] examined the trait evaluation of an actor who had helped a person in a scenario in which no information was given regarding whether the actor had received prior help from the person being helped (no prior information condition), in addition to trait evaluations for the reciprocal prosocial actor and the unilateral prosocial actor, who were presented in the OR condition and the EX condition in the present study. The results of Futamura [20] showed that the trait evaluations of kindness and niceness for the no-prior-information actor and the unilateral actor were higher than those for the reciprocal actor, although there was no difference between the no-prior-information actor and the unilateral actor. This result indicates that the extraordinary prosocial behavior used in this study was not particularly *extreme* extraordinary behavior.

One of the limitations of this study was that the control of the valence in each condition was insufficient in Study 2. The conditions in Study 2 were set by the pattern of behaviors as a 2 (ordinary prosocial behavior: engaging or non-engaging) × 2 (extraordinary prosocial behavior: engaging or non-engaging) combination to examine the perceived value of ordinary prosocial behaviors and extraordinary prosocial behaviors themselves. However, each condition in Study 2 included a different amount of positive or negative valence expressions, and we could not differentiate the effect of engaging or not engaging in ordinary and extraordinary prosocial behaviors from the effect of positive or negative valence included in each condition. However, Study 2 also revealed results that could not be explained only by the effects of positive or negative valence. For example, although the valence for the OR-ex and or-EX conditions was the same, there were differences in the evaluations of the OR-ex and or-EX actors’ morality. Thus, Study 2 provides meaningful suggestions for understanding the functions of ordinary and extraordinary prosocial behaviors. However, there is a need for further research to examine the cognition of ordinary and extraordinary prosocial behaviors using more elaborate designs to control valence and to create an exact and detailed understanding of the social functions of such behaviors.

Another limitation was that the behavior tendency in the or-EX condition, in which the actors do not engage in ordinary prosocial behavior but in extraordinary prosocial behavior, is less common and may seem somewhat unnatural. As indicated by Skowronski and Carlston [18], the current paper’s Study 1 has shown that people perceive that extraordinary prosocial actors are also highly likely to engage in ordinary prosocial behaviors. According to this result, it is possible that participants had difficulty to image the target clearly in the or-EX condition compared to the participants in other conditions and that the unnaturalness of the condition affected the results of Study 2. Although this manipulation in Study 2 was set to examine how the effects of ordinary and extraordinary prosocial behaviors on trait evaluation are different between the case of the actor engaging in and of the actor not engaging in the other behavior, future research needs to examine the value of each ordinary and extraordinary prosocial behaviors by using situations that are more familiar to the participants.

A further extension of the present study is to examine the effects of individual and cultural factors on the evaluation of ordinary and extraordinary prosocial behaviors. Klein et al. [9] examined the trait evaluations for a selfish actor, a fair actor, and a generous actor using a dictator game in a seven-country experiment. The result showed that the fair actor was evaluated more positively than the selfish actor in all seven countries. However, there was a difference in the evaluations of the fair and generous actors depending on the country; the generous actor was evaluated higher than the fair actor only in one country (China), and the participants of one country (Turkey) evaluated the fair actor rather higher than the generous actor. Thus, the evaluation or value of extraordinary prosocial behavior appears to depend both on the individual and the culture; it can sometimes be difficult to judge whether to engage in extraordinary prosocial behaviors in daily life. The role of both individual and cultural factors in evaluating ordinary and extraordinary prosocial behaviors should be the subject of future research.
